# Increasing rates of *erm*(B) and *erm*(N) in human *Campylobacter coli* and *Campylobacter jejuni* erythromycin-resistant isolates between 2018 and 2023 in France

**DOI:** 10.1128/aac.01668-24

**Published:** 2024-12-31

**Authors:** Quentin Jehanne, Lucie Bénéjat, Astrid Ducournau, Johanna Aptel, Marie Pivard, Léo Gillet, Marine Jauvain, Philippe Lehours

**Affiliations:** 1 National Reference Centre for Campylobacters & Helicobacters, Bordeaux, France; 2 University of Bordeaux, Inserm, UMR 1312, BRIC, BoRdeaux Institute of onCology 640680 , Bordeaux, Nouvelle-Aquitaine, France; Johns Hopkins University School of Medicine, Baltimore, Maryland, USA

**Keywords:** *Campylobacter*, resistance, NGS, macrolide, methyltransferase

## Abstract

Macrolides are the first-line compounds used for the treatment of campylobacteriosis. Macrolide resistance remains low in France, with mutations in *23S rDNA* being the main associated resistance mechanism. However, two erythromycin methyltransferases have also been identified*: erm*(B), which is mainly described in animal reservoirs, and *erm*(N), which is strictly described in humans. In France, between 2018 and 2023, erythromycin-resistant *Campylobacter* species strains were systematically sequenced and analyzed *via* an in-house bioinformatics pipeline, leading to the identification of the resistomes, MLST and cgMLST, as well as the characterization of the source of contamination. In this study, the genomes of 280 erythromycin-resistant strains were sequenced over a 6-year period. The identification of erythromycin-associated resistance markers revealed a predominance of *23S rDNA* mutations, in 90% of cases, but also *erm*-type methyltransferases in 10% of cases: 75% for *erm*(N) and 25% for *erm*(B). Over this period, an important increase in the rate of *erm*-positive isolates was observed: 2% in 2018 compared with 13% in 2023, with 10% for *erm*(N) and 3% for *erm*(B). *erm*(N) has been found exclusively within a CRISPR–Cas9 operon, whereas *erm*(B) has been found within diverse types of resistance genomic islands. Each *erm*(N)- or *erm*(B)-positive isolate had at least two other resistance markers (mostly ciprofloxacin, tetracycline, or ampicillin) and often carried aminoglycoside-associated resistance genes. The majority of the *erm*-positive isolates were obtained from chicken. The increasing rates of *erm*-positive and multiresistant isolates make the monitoring of erythromycin-resistant *Campylobacter* strains, specifically within the chicken meat production, a topic of serious importance.

## INTRODUCTION

*Campylobacter* infections are the leading cause of bacterial gastroenteritis in Europe (1). Symptoms of *Campylobacter* infections are mainly acute gastroenteritis, which is usually mild and self-limiting within a week (2). Complications associated with *Campylobacter* infections are rare (e.g., death in less than 0.1% of cases) and occur mainly in frail individuals (newborn, elderly, or immunocompromised patients). In such cases, the first-line treatment involves the administration of a macrolide (e.g., azithromycin) (3). In France, epidemiological surveillance of *Campylobacter* infections is based on a network of clinical laboratories sending their isolates to the National Reference Center for Campylobacters and Helicobacters (NRCCH) (www.cnrch.fr), as well as on mandatory reporting of collective food poisoning outbreaks in which *Campylobacter* is the confirmed pathogen. However, cases of infections reported by these two surveillance systems represent only a fraction of the cases that actually occur. In France, the average annual number of symptomatic cases of *Campylobacter* infections has been estimated at 493,000 (90% confidence interval (CI): 273,000–1,080,000), of which 392,000 are thought to have been infected through food transmission. *Campylobacter* is responsible for 26% of the estimated total number of foodborne infections and 31% of the hospitalizations associated with these infections (4).

At the NRCCH, all *Campylobacter* isolates collected between 2018 and 2023 were identified *via* MALDI-TOF mass spectrometry, and the antimicrobial resistance (AMR) was tested. Particular attention was given during this period to the evolution of macrolide resistance, which remained below 1% and 10% for *C. jejuni* and *C. coli*, respectively. These results are comparable to those from other European countries, with the exception of human clinical isolates of *C. jejuni* in Spain, where the level of resistance to erythromycin is greater than 10%, as well as greater than 55% for *C. coli i*n Portugal (European Centre for Disease Prevention and Control data: https://www.ecdc.europa.eu/en/campylobacteriosis/antimicrobial-resistance).

Macrolides, such as erythromycin, bind to the 50S subunit of the ribosome and inhibit protein synthesis. Mutations in *23S rDNA* that block this molecular binding are associated with macrolide resistance, and the most frequent mutations are A2074C, A2074G, or A2075G, with the A2074T mutation rarely detected (5). They are generally present within all three copies of the *23S rDNA* gene and induce a high level of resistance to erythromycin, with minimum inhibitory concentrations (MICs) over 128 mg/L; to other macrolides (e.g., tylosin, azithromycin, clarithromycin, and telithromycin); and to lincosamides (e.g. clindamycin). In 2014, *erm*(B), a novel gene encoding an rRNA methyltransferase in *Campylobacter* isolates from food animals (pigs, chickens, and ducks) was described (6). *erm*(B) is associated with a very high level of resistance to erythromycin (MICs over 512 mg/L), lincosamide, and streptogramin B (7). *erm*(B) can be carried by transferable plasmids or by horizontal gene transfer and is found within multidrug resistance genomic islands (or MDRGI), which includes genes such as *tet*(O) for tetracycline resistance or *APH*(2″) for gentamicin resistance. This first methyltransferase is the most represented in *Campylobacter*, notably in Asian countries such as China, where it was first identified in 2008 (6). *erm*(B) was rarely described in the rest of the world, in Belgium in 2019 (8), in Spain in 2017 (9), in the United States in 2018 (10), and in Australia in 2020 (11). In a previous study, all erythromycin-resistant isolates from the NRCCH since 2016 were tested for *erm*(B) by PCR, and the first two clinical *erm*(B)-positive *C. coli* isolates from France were identified, one from 2017 and the other from 2018 (12). Moreover, a novel methyltransferase called *erm*(N)*,* inserted within the CRISPR repetitive sequences of the CRISPR–Cas9 operon, has also been described in *C. coli* clinical isolates from France and Quebec (12, 13). It is not transferable by natural conjugation and is associated with heterogeneous levels of resistance to erythromycin (MICs ranging from 16 to 512 mg/L)(12). In addition to these various modifications of macrolide ribosomal targets, the efflux likely plays a minor role in macrolide resistance, as do various mutations, insertions, or deletions in the ribosomal proteins L4 and L22, which are encoded by the *rplD* and *rplV* genes, respectively (14–16).

The aim of the present study was to evaluate the mechanisms of resistance to erythromycin in France during the 6-year period from 2018 to 2023 *via* a systematic sequencing strategy for *in vitro* erythromycin-resistant strains. Here, we demonstrate an increase in *erm*(B) and especially *erm*(N) methyltransferases over this period in *C. coli* and *C. jejuni*.

## MATERIALS AND METHODS

### Selection and isolation of clinical erythromycin-resistant isolates

A total of 280 clinical isolates of either *C. coli* (*n* = 240, 85.7%) or *C. jejuni* (*n* = 40, 14.3%) that were detected *in vitro* as erythromycin-resistant were included in the present study (complete data table available in Table 1 ). Our data consist of every single erythromycin-resistant isolate from 2018 to 2023 (6-year period) isolated from stool (*n* = 263, 93.9%), blood (*n* = 16, 5.7%), and gastric biopsy (*n* = 1*,* 0.4%) samples and sent from various laboratories across France to the French National Reference Center for Campylobacters and Helicobacters (NRCCH) (www.cnrch.fr). Each metropolitan French region was involved. In fact, 36.4% of the studied isolates were obtained from patients in the southern part of France (*n* = 102), 23.9% from the eastern region (*n* = 67), 12.1% from around Paris (*n* = 34), 11.8% from the northern region (*n* = 33), 9.3% from the western region (*n* = 26), and 6.1% from the central region (*n* = 17). Only one isolate was sampled from the overseas territory (CNRERY-01526, La Réunion Island) (0.4%). The mean age and sex ratio (male/female) of the included patients were approximately 42 ± 27.2 years and 1.5, respectively. Each *C. coli* and *C. jejuni* strain was initially isolated on a Columbia blood agar (CBA) plate with 5% sheep blood (Thermo Fisher Scientific, MA) and incubated at 37°C in a jar. An anoxomat microprocessor (Mart Microbiology BV, Lichtenvoorde, The Netherlands) created a microaerobic atmosphere of 80 to 90% N_2_, 5 to 10% CO_2_, and 5 to 10% H_2_.

**TABLE 1:** Proportion of resistant isolates (%) on the basis of AST and the presence of antimicrobial resistance-associated genes or mutations*^a^*

Group	No. of isolates (% of total data set)	AMP	CIP	TET	GEN	KAN	STR	SPC	CHL	LIN
Full data set of ERY-R isolates	280 (100)	56.8	92.1	88.9	13.6	24.6	56.8	25.7	1.1	1.1
*C. coli*	240 (85.7)	60.0	91.2	95.4	15.4	27.5	64.6	29.6	1.2	1.2
*C. jejuni*	40 (14.3)	37.5	97.5	50.0	2.5	7.5	10.0	2.5	0.0	0.0
23S-mutated isolates	252 (90.0)	53.6	91.3	87.7	12.7	24.2	56.3	23.4	0.8	0.8
erm-positive isolates	28 (10.0)	85.7	100.0	100.0	21.4	28.6	60.7	46.4	3.6	3.6
erm(N)-positive isolates	21 (7.5)	95.2	100.0	100.0	19.0	28.6	47.6	28.6	0.0	0.0
erm(B)-positive isolates	7 (2.5)	57.1	100.0	100.0	28.6	28.6	100.0	100.0	14.3	14.3

^*a*^ Phenotypic antimicrobial susceptibility testing (AST) highlighted in gray (AMP: ampicillin; CIP: ciprofloxacin; TET: tetracycline; GEN: gentamicin) was performed via the disk diffusion method and verified *in silico* based on the identification of AMR-associated mechanisms using BLASTN and multiple gene and mutation databases (NCBI, CARD, ResFinder, and the in-house NRCCH resistance database). The remaining antimicrobial resistances (KAN: kanamycin; STR: streptomycin; SPC: spectinomycin; CHL: chloramphenicol; LIN: lincomycin) were determined only via *in silico* analyses. The values are highlighted in bold when one-third of the isolates are resistant.

### Bacterial identification and antibiotic susceptibility testing

Bacterial species were identified from pure cultures *via* matrix-assisted laser desorption/ionization time-of-flight mass spectrometry method, as previously described (17). Antimicrobial susceptibility testing (AST) to erythromycin and four additional antimicrobials (ampicillin, ciprofloxacin, gentamicin, and tetracycline) was assessed *via* the disk diffusion method (DD) based on the CASFM/EUCAST 2022 recommendations for *Campylobacter* species (18). Precisely, an inoculum at 0.5 McFarland standard of pure *Campylobacter* was subcultured on Mueller–Hinton (MH) agar supplemented with 5% defibrinated horse blood (MH-F) and 20 mg/L nicotinamide adenine dinucleotide (β-NAD) (bioMérieux, Marcy l’Etoile, France), and incubation was performed for 48 hours in a microaerobic environment at 37°C. The inhibition zone diameters were measured *via* the SIRscan Auto (i2A, Montpellier, France) automatic system, and the data were read based on the CASFM/EUCAST 2022 data (18). Additionally, *C. jejuni* reference strain CCUG 11284 was used as a quality control strain.

### Minimum inhibitory concentrations of *erm*-positive and *23s rDNA*-mutated isolates

Erythromycin MICs were determined on MH-F for each isolate included in the present study *via* Etest strips (bioMérieux). Following 48 hours of incubation, the point at which the zone of growth inhibition intersected the strip was recorded as the MIC in mg/L. The reference strain *C. jejuni* ATCC 33560 was used as a quality control strain, according to the CASFM/EUCAST recommendations (18). From a selection of all *erm*-positive isolates identified in this study and an equivalent number of *23S rDNA*-mutated *Campylobacter* isolates, erythromycin MICs were verified *via* the agar dilution method. Briefly, MH-F agar plates were prepared with or without erythromycin. A stock solution of 81.92 mg/mL erythromycin (from 1 g of erythromycin lactobionate in 12.2 mL, Pro Concepta Zug AG, Switzerland) was prepared in sterile water. Then, adapted dilutions were prepared to obtain agar plates containing concentrations ranging from 8.192 µg/mL to 4 µg/mL. The inoculation was then performed with a Steers apparatus (Masturi Dot, MAST Diagnostic, Amiens, France). The plates were incubated for 48 hours at 37°C in jars *via* an Anoxomat microprocessor. The MICs were determined by two independent readers as the lowest concentration (µg/mL) of the drug that inhibited the growth of the strain studied. Three *erm*(N)*-*positive *C. coli* isolates (CNRERY-00683, CNRERY-00695, and CNRERY-00859) and two *erm*(B)-positive *C. coli* isolates (CNRERY-00836 and CNRERY-00883) from a previous study (12), as well as two susceptible clinical isolates (one *C. jejuni* and one *C*. *coli*, not included in Table S1), were used as quality control strains. The results are displayed in box plots using GraphPad Prism 8.4.3 (GraphPad Software, Inc., San Diego, CA, United States). The Mann–Whitney test was used as a nonparametric test to compare erythromycin MICs between *23S rDNA*-mutated and *erm*-positive isolates. Differences were considered significant when *P* was less than 0.05.

### Whole-genome sequencing and assembly of *Campylobacter* isolates

To determine erythromycin-associated mechanisms, whole-genome sequencing (WGS) was performed on previous pure cultures of each isolate. DNA was extracted *via* the MagNA Pure 6 DNA and viral NA SV kit, which uses bacterial lysis, and the MagNA Pure 96 system (Roche Applied Science, Manheim, Germany). Paired-end sequencing was performed *via* Illumina technology. Multiple sequencers were used from 2018 to 2023: an Illumina HiSeq 4000 (*n* = 33), an Iseq 100 (*n* = 42), and a NovaSeq 6000 (*n* = 205). The raw sequencing data (.fastq) were cleaned using Sickle v1.33 (19) and the genomes were *de novo-*assembled using SKESA v2.5.1 (20).

### Whole-genome analyses

Species were confirmed *via* the molecular average nucleotide identity (ANI) method using FastANI v1.33 (21): a threshold of ⩾95% validated species identification. Sequence type (ST), clonal complex (CC), and core-genome MLST were identified using PubMLST *C. jejuni* and *C. coli* databases (cgMLST *Campylobacter* scheme v2.0) (22). From the PubMLST alignment output, the cgMLST tree was displayed using MEGA software v11 (23), combined with the iTOL online tool v6 (24). Antimicrobial resistance (AMR)-associated mechanisms were determined *via* the Blastn command line tool v2.15.0+ (25) combined with multiple genes, proteins, and mutations databases: the NCBI, CARD, and ResFinder databases as well as the in-house NRCCH *Campylobacter* resistance database. Source attribution within the chicken, ruminant, and environment reservoirs for *C. jejuni* and the chicken, ruminant, and pig reservoirs for *C. coli* was estimated using STRUCTURE (26) combined with host-segregating genes (27, 28) and mutations (29), respectively. Finally, genome annotations were performed *via* Prokka v1.14.5 (30), and plasmid DNA was predicted using the RFPlasmid v1.0 tool (31).

### *Erm*(N) and *erm*(B) genomic region characterization

The genomic region surrounding the *erm*(N) or *erm*(B) genes was extracted using the Blast graphical online tool (32) or reconstructed manually for incomplete assemblies. For the *erm*(N) region, it consists of three genes before and one gene after the methyltransferase (−5,000/+2,100 nucleotides). For *erm*(B), each gene before and after was displayed as soon as it was associated with antimicrobial resistance or virulence (−6/+6 genes on average). Moreover, the raw sequencing data of each *erm*(B)-positive isolate were aligned to 11 different types of MDRGI-containing *erm*(B) previously described (33, 34) using bwa v0.7.17 (35) and samtools 1.19.2 (36), and the highest coverage score indicated the most likely MDRGI type.

## RESULTS

### Genomic characterization of *C. jejuni* and *C. coli* isolates

**Fig 1:**
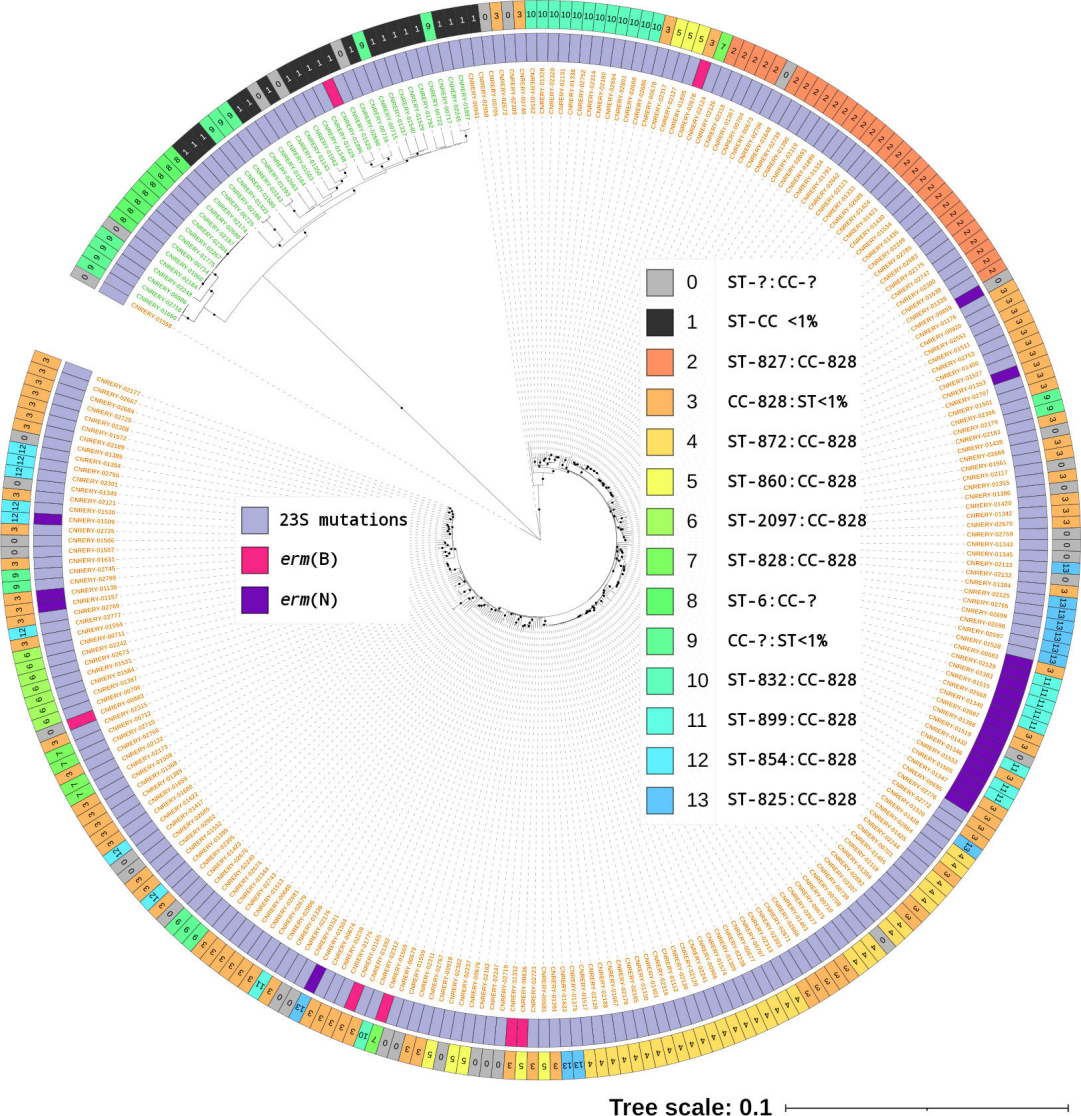
Core-genome MLST tree of all 280 studied *C. coli* and *C. jejuni* clinical isolates. Core-genome profiles were identified *via* the *Campylobacter* scheme v2.0 from PubMLST, and the tree was displayed using MEGA software combined with the iTOL online tool. *C. coli* isolates are highlighted in orange, whereas *C. jejuni* isolates are highlighted in green. Various STs and CCs were found, and their combinations were attributed to a specific color. Furthermore, “**ST-?**” or “**CC-?**” annotations were used to display undefined STs or undefined CCs, respectively, and “**<1%**” annotation was used to display STs or CCs with fewer than 1% of the studied isolates, being unique STs or CCs identified in this study. Dots on branches indicate a bootstrap score of 100%.

Globally, 90% ±5.3% of the raw read data of studied isolates were mapped against their reference genome, and *de novo-*assembled genomes were at 1,738,211 bp of size ±122 kbp, 39,99 contigs ±54.7, and a GC% of 30.84% ±1.7% ( Table S1). Erythromycin-resistant *C. jejuni* and *C. coli* isolates were categorized into various sequence types by clustering analysis, regardless of the mechanism of resistance involved (Fig. 1). In fact, the two most predominant STs were ST-827 and ST-872 with 30 isolates each (21.4% of the total data set), followed by ST-832 with 13 isolates (4.6% of the total data set). However, a total of 34 isolates were found with undefined STs, which represented 12.1% of the data set (30 *C*. *coli* and 4 *C*. *jejuni* isolates). Among the *23S*-mutated isolates, ST-827 and ST-872 were also the main clusters (23.8% with 60 isolates), whereas ST-899/CC-828 was predominant among *erm*(N)-positive *Campylobacter*, with eight isolates (38.1%), followed by ST-9840/CC-828 with four isolates (19%). Regarding *erm*(B), each positive isolate (*n* = 7) possessed a unique combination of ST/CC. In general, CC-828 represented 71.8% of the total data set and was the main complex among *23S*-mutated and *erm*-positive isolates, with 177 and 24 isolates, respectively.

STRUCTURE analysis of the hypothetical source of contamination revealed a large number of strains that were assigned to the chicken reservoir, which represented 66.8% of the total data set (187 isolates, 166 *C*. *coli* and 21 *C*. *jejuni*), followed by the pig reservoir with 73 isolates (26.1%, only *C. coli* isolates). No reservoir was specific to a resistance mechanism or sequence type.

### Antimicrobial resistance profiles

Each strain had additional resistance markers in addition to erythromycin. Among these erythromycin-resistant isolates, 56.8% of the total data set was also resistant to ampicillin (159 isolates, 144 *C*. *coli* and 15 *C*. *jejuni*), mainly associated with a mutation in the promoting region of their beta-lactamase (G57T for 152 isolates and A61G for one isolate) (37) or with an undescribed promoting region (six isolates, two *C*. *coli* and four *C*. *jejuni*) (Table S1). Among all the ampicillin-resistant isolates, *bla*_oxa-193_ was the main beta-lactamase identified with 93 isolates (58.5%), followed by *bla*_oxa489_ with 49 isolates (30.8%). Resistance to ciprofloxacin was also very common, with 92.1% of isolates (258 isolates, 219 *C*. *coli* and 39 *C*. *jejuni*) showing amino-acid substitutions in the GyrA protein sequence, mainly T86I (249 isolates) alone or with D90Y (four isolates, three *C*. *coli* and one *C*. *jejuni*) or D90N (eight isolates, four *C*. *coli* and four *C*. *jejuni*). The mutation T86R was also detected among nine *C*. *jejuni* isolates. A total of 249 isolates (88.9%) also expressed a tetracycline resistance gene, mainly *tet*(O) (168 isolates, 156 *C*. *coli* and 12 *C*. *jejuni*), *tet*(O-32-O) (48 isolates, 41 *C*. *coli* and seven *C*. *jejuni*), and *tet*(O-M-O) (28 isolates, 27 *C*. *coli*, and one *C*. *jejuni*). A total of 145 strains (51.8%) were multiresistant to erythromycin, ampicillin, ciprofloxacin, and tetracycline. One *C. coli* strain had nine resistance markers (CNRERY-01521): erythromycin, ampicillin, ciprofloxacin, tetracycline, gentamicin, lincomycin, kanamycin, streptomycin, and spectinomycin. Aminoglycoside resistance was also considerable. Gentamicin resistance was detected in 13.6% of the isolates (38 isolates, 37 *C*. *coli* and one *C*. *jejuni*), with a majority of *aph2’’* encoding genes (*n* = 31, 81.6% of all gentamicin-resistant isolates). Resistance markers for kanamycin *aph*(3*’*)-IIIa were found among 66 *C*. *coli* and three *C*. *jejuni* (27.5% of the total data set), *ant6* types and *sat-4* streptomycin resistance-associated genes were found among 155 *C*. *coli* and four *C*. *jejuni* (56.8% of the total data set), and *ant9 or spw* spectinomycin resistance genes were found among 71 *C*. *coli* and one *C*. *jejuni* (25.7%). Chloramphenicol resistance (*cat* gene) was detected in three *C*. *coli* isolates, as the lincosamide resistance-associated gene *lnuC*. Using RFPlasmid, a putative plasmid was identified in 9.6% of all the isolates (23 *C*. *coli* and four *C*. *jejuni*), encoding from one to three resistance genes, mainly *tet*(O), a*ph3’’-IIIA*, *ant6,* and *cat* genes.

### Erythromycin resistance evolution and mechanism proportions

**Fig 2:**
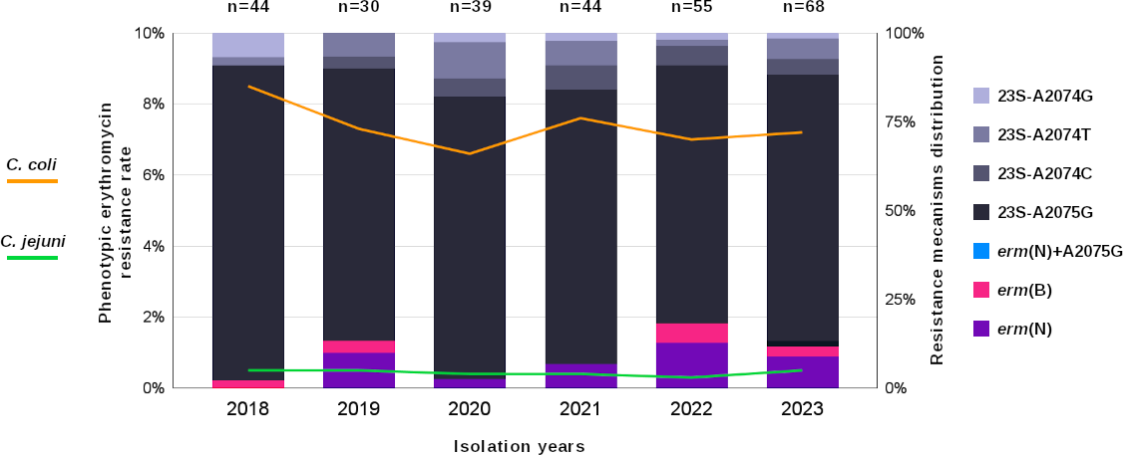
Evolution of *C. jejuni* and *C. coli* erythromycin-resistant clinical isolates between 2018 and 2023 in France, with the associated resistance mechanism proportions. The left y-axis displays erythromycin resistance rates in France between 2018 and 2023; orange represents *C. coli* clinical isolates (*n* = 1,077 isolates tested per year on average, data not included), and green represents *C. jejuni* clinical isolates (*n* = 6,870 isolates tested per year on average, data not included). These data are based on NRCCH annual reports (www.cnrch.fr/). The right y-axis shown with stacked bars indicates the proportion of each resistance mechanism associated to erythromycin for each year and the isolates that were sequenced in the present study. The total number of erythromycin-resistant isolates per year is indicated above the corresponding stacked bar.

In the present study, the evolution of erythromycin-resistant *Campylobacter* isolates was analyzed over a period of 6 years in France. The resistance rates of *C. jejuni* and *C. coli* remained stable, as displayed in (Fig. 2). However, *C. coli* isolates displayed greater resistance to erythromycin than did *C. jejuni*, with an average resistance of 7.4% against 0.4% for *C. jejuni*. Important divergence between the two species was also observed regarding the presence of *23S* mutations and *erm* expression. Among the 28 *erm-*positive isolates, 27 were *C. coli* (96.4%), with 21 *erm*(N) isolates and six *erm*(B) isolates. Only one *C. jejuni* isolate expressed *erm*(B) (CNRERY-01896). This last isolate was also resistant to ampicillin, ciprofloxacin, tetracycline, kanamycin, streptomycin, and spectinomycin. Among these *erm-*positive isolates, 19 (68%) were of chicken origin (13 *erm*(N) and six *erm*(B) isolates), and eight were of pig origin (32%). The single *erm*(B)-positive *C. jejuni* isolate identified from the chicken reservoir was an ST-10025/CC-353 strain.

In contrast, among the 252 isolates with *23S rDNA* mutations (90% of the total data set), 84.5% were *C. coli* (*n* = 213), whereas 15.5% were *C. jejuni* (*n* = 39). The main *23S rDNA* found in *C. coli* was A2075G (97.2% with 207 isolates), whereas the distribution was more diverse in the *C. jejuni* isolates, with 38.5% for A2074T (15 isolates), 28.2% for A2075G (11 isolates), 28.2% for A2074C (11 isolates), and finally 5.1% for A2074G (two isolates).

Over the years, an increase in *erm*-expressing *Campylobacter* isolates was observed over *23S rDNA*-mutated isolates, whereas in 2018, 98% of the erythromycin-resistant *Campylobacter* had mutations in the *23S rDNA* against only 2% of the *erm*-expressing isolates, and in 2022 and 2023, a sevenfold to ninefold increase was observed: 18% in 2022 and 13% in 2023 of *Campylobacter* expressed either *erm*(N) or *erm*(B), whereas 82% in 2022 and 87% in 2023 had *23S rDNA* mutations. Interestingly, in 2023, one *C. coli* isolate had an A2075G mutation, in addition to *erm*(N). While the number of erythromycin-resistant isolates remained stable in 2020 and 2021, few *erm*-positive isolates were detected. In addition to the period coinciding with the SARS-CoV-2 outbreak, no convincing element could explain these lower rates.

**Fig 3:**
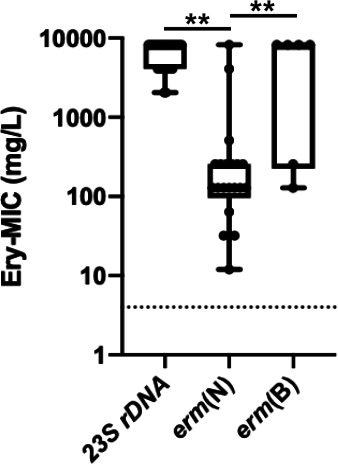
Erythromycin minimum inhibitory concentration distributions from the agar dilution method. Boxplots were drawn using GraphPad Prism from MICs (mg/L) from a selection of all *erm*-positive isolates (*erm*(N): *n* = 7; *erm*(B): *n* = 21), and 33 *23S rDNA*-mutated isolates (A2074T: *n* = 9; A2074C: *n* = 8; A2074G: *n* = 7; A2075G: *n* = 9). A nonparametric Mann–Whitney test revealed a significant difference between *23S*-mutated isolates and *erm*-positive isolates (*23S* vs. *erm*(N): *P* < 0.001**; *23S* vs. *erm*(B): *P* = 0.65 *ns; erm*(N) vs. *erm*(B): *P* = 0.008**).

In terms of erythromycin MICs, all *23S rDNA*-mutated isolates (either *C. coli* or *C. jejuni*) had MICs greater than 256 mg/L according to the Etest (with MICs ranging from 2028 to >8192 mg/L *via* the agar dilution method), whereas *erm*-positive isolates had significantly lower MICs ranging from 16 to over 256 mg/L when the Etest MICs were considered (with MICs ranging from 12 to >8,192 mg/L according to the agar dilution method). Interestingly, MICs were greater in *erm*(B)-positive strains than in *erm*(N)-positive strains. The MICs were significantly different between the *23S rDNA*-mutated and *erm*(N) isolates, but not between the *23S rDNA*-mutated and *erm*(B) isolates (Fig. 3).

### Erythromycin resistance methyltransferase genomic regions

**Fig 4:**
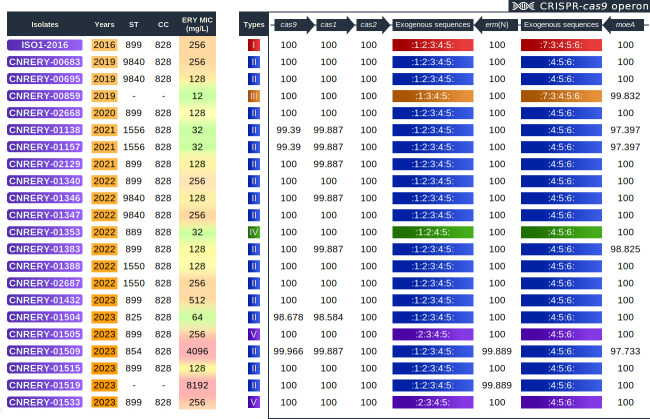
CRISPR–Cas9 operons of each *erm*(N)-positive *C. coli* clinical isolate. CRISPR–Cas9 regions were extracted from assembly data between the *cas9* and *moaE* genes. Various types of CRISPR arrays were identified and are indicated in colored boxes as follows: type I in red, II in blue, III in orange, IV in green, and V in purple. The number within each type indicates exogenous sequences, and “:” indicates the *C. coli* palindromic repeat sequence “ATTTTACCATAAAGAAATTTAAAAAGGGACTAAAA.” The exogenous sequences are as follows: 1 = CCTATTGCAACCCTTGTTTCACGACTATAA; 2 = TTTGCAAGATAGTGATTTAAGAGATGCTTT; 3 = AAGTTTTGAAACAAGAGTGTATTATGATTA; 4 = CACCCTTCCAAAAGGGTGGAGAAGGGTTTA; 5 = GTTTTTATTTGTGGTTATAAAATAAAAAAG; 6 = TTCATAGCATCTTGCGAGCTTTTAAAGGCA; 7 = TTGCAAGATAGTGATTTAAGAGATGCTTT. The sequences for *cas9*, *cas1*, *cas2*, *erm*(N), and *moeA*, as indicated by percentages in the figure, were almost identical among all the isolates. The erythromycin MICs highlighted here are those obtained *via* the agar dilution method. The isolate “ISO1-2016”, not included in the present study, is used here as an example of a type I *erm*(N) isolate, as previously described (12).

In the present study, the *erm*(N) and *erm*(B) genes were uniquely found within chromosomal regions. *erm*(N), inserted within CRISPR-Cas9 as previously described (12), was almost fully conserved among each isolate (Fig. 4). The surrounding genes (*cas9*, *cas1*, *cas2,* and *moeA*) as well as intergenic regions were also identical, with few nucleotide variations. However, notable differences were observed regarding the exogenous sequences within the CRISPR arrays. Each *erm*(N) locus was attributed to a type depending on the exogenous DNA sequences found within the CRISPR array. A total of seven different exogenous sequences (1 to 7 as follows) were identified, and their different combinations allowed the determination of five types of CRISPR-Cas9-*erm*(N) regions: type I: 12345-*erm*(N)−73456; type II: 12345-*erm*(N)−456; type III: 1345-*erm*(N)−73456; type IV: 1245-*erm*(N)−456; and type V: 2345-*erm*(N)−456. Among the 21 isolates, 17 possessed the type II CRISPR–Cas9 operon, two were associated with type V, one was associated with type III, and the last one with type IV. Interestingly, the CNRERY-00859 isolate harboring a type III CRISPR–Cas9 operon had the lowest MIC (12 mg/L as determined by the agar dilution method), which is in line with our previous findings (12). Otherwise, no clear correlation between MICs and CRISPR–Cas9 operon types was identified.

**Fig 5:**
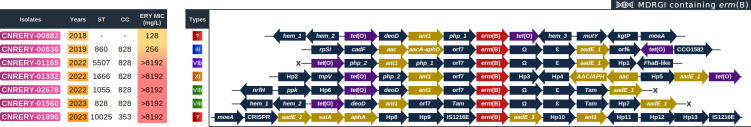
Chromosomal multidrug resistance genomic islands (MDRGI) of each *erm*(B)-positive isolate. The MDRGI was extracted from the assembly data at an average of −6/+6 genes surrounding *erm*(B) (in red). Genes annotated as *tet*(O) using Prokka are displayed in purple, and other resistance genes are in yellow. The remaining genes are not related to AMR or correspond to hypothetical genes (*Hp* = hypothetical protein; *hem* = bacteriohemerythrin; *php* = phosphorylase; *tam* = trans-aconitate 2-methyltransferase; IS1216E = transposase). MDRGI types were defined based on the alignment of raw sequencing data against 11 types defined in previous publications (33, 34). Undefined types are indicated as “?” red boxes. The erythromycin MICs highlighted here are those obtained *via* the agar dilution method.

On the other hand, *erm*(B) was found to be inserted within various types of multidrug resistance genomic islands (Fig. 5). The raw read data of *erm*(B)-positive isolates revealed that the CNRERY-00836 and CNRERY-01165 isolates presented a type III and VIb MDRGI, respectively, with 100% coverage. Isolates CNRERY-02678 and CNRERY-01560 MDRGIs were type VIII with 96.9% and 97.58% coverage, respectively, and isolate CNRERY-01332 most likely displayed a type XI with 80.13% coverage. However, we were unable to precisely identify which MDRGI type isolates CNRERY-00883 and CNRERY-01896 belong to. In fact, the coverage scores were too low, and no similar resistance island was found on the basis of previous publications (33, 34). Moreover, *erm*(B)-positive isolates carried multiple copies of *tet*(O), which prevents the proper assembly of such chromosomal regions. Therefore, MDRGI-type identification may yield inconsistent results.

## DISCUSSION

In this study, we aimed to characterize *via* WGS the molecular mechanisms associated with erythromycin resistance among *C. jejuni* and *C. coli* strains isolated from clinical cases in France from 2018 to 2023. This study included clinical isolates originating from all regions of France and therefore presented no geographical selection bias. Although *23S* mutations prevailed among these isolates, we detected a noticeable increase in the proportion of *erm*-positive clinical isolates from 2020 onward, with *erm*(N) methyltransferase predominating over *erm*(B). These particular strains of *Campylobacter*, mostly *C. coli*, presented a variety of STs and CCs as well as multiresistant profiles.

According to the last ECDC report for campylobacteriosis (38), erythromycin resistance in *C. jejuni* and *C. coli i*solates obtained from humans significantly increased in some countries, such as Spain, but significantly decreased in others, such as Norway and the United Kingdom. In France, the situation has remained stable over the last 10 years (NRCCH annual reports, www.cnrch.fr/) where, despite the emergence of *erm* genes between 2018 and 2023, the extent of macrolide resistance in *C. jejuni* and *C. coli* has not increased. Nevertheless, the ECDC recommends analyzing any highly resistant or MDR isolate *via* molecular methods such as whole-genome sequencing (WGS) to precisely monitor potential outbreaks of concerning strains. The present study is therefore in line with these recommendations.

Erythromycin resistance in campylobacters in Europe is almost entirely acquired by *23S rDNA* mutations. While in the present study the A2075G mutation is predominant among *C. jejuni* isolates, we also found a variety of genotypes over this 6-year period, specifically at position 2074 (A2074G, A2074C, and A2074T). These results differ greatly from what we can observe in China, where A2075G may sometimes be the only mutation identified, whether for *C. jejuni* or *C. coli* (39). The ECDC report also mentioned that the recent discovery of the methyltransferase *erm*(B) is a matter of concern. Widely distributed in Gram-negative but also Gram-positive bacteria (40), this gene is more frequently observed in *C. coli* than in *C. jejuni*, and in the animal food chain more than in humans, which is specifically concerning in China, and in chicken meat (34,41–43). The idea that poultry reservoirs spread multiresistant strains such as *erm*-positive *Campylobacter* isolates is now a worldwide issue. As a matter of fact, *erm*(B) can now be detected in various countries: three *erm*(B)-positive isolates (one *C. coli* and one *C. jejuni*) from pastured poultry farms in the United States described in 2016 (44), two *C. coli* isolates from native chickens in Thailand in 2022 (45), 14 isolates of *C. jejuni* from slaughtered broiler chickens in South Africa between 2017 and 2018 (46), and 3.2% of *erm*(B)-positive isolates (12 *C*. *coli* and three *C. jejuni*) detected in Taiwan from 2016 to 2019 (47). In eggs from a laying hen farm in Tunisia between 2017 and 2018, the *erm*(B) gene was detected at concerning rates of 48.38% and 64.15% for the *C. jejuni* and *C. coli* isolates, respectively (48). In Europe, however, *erm*(B) has been rarely reported, except in *C. coli* isolates from a broiler strain in Belgium (*n* = 1) and from broilers and turkeys in Spain (*n* = 2) (5, 8). Moreover, transmission to humans is becoming significant, especially in Asia. A recent study revealed an important proportion of *erm*(B)-expressing isolates in the clinical context in Shanghai between 2012 and 2019, with 50% of the studied *C. coli* strains expressing this methyltransferase (39). In Taiwan in 2021 and 2022, 60.5% of *C. coli* from human campylobacteriosis cases from collaborative hospitals were *erm*(B)-positive and 3.4% were *C. jejuni* (47). In Europe, clinical *erm*(B)-positive *Campylobacter* is uncommon and has been reported only once by our laboratory (12). This is consistent with the low rates of positive isolates found within food animals and may explain the low rates of the spread of this resistance mechanism in France. As suggested by a previous study, *erm*(B) transmission between *Campylobacter* bacteria may occur because of a putative circular MDRGI intermediate formed by recombination between the *tet*(O) genes (34). Our study is in line with this hypothesis since we did observe two copies of *tet*(O) among almost every *erm*(B)-positive isolate, which resulted in truncated or circular contig assemblies at these locations. As previously described (49), the presence of two IS1216E transposases within one *erm*(B)-positive isolate (CNRERY-01896) also indicates putative recombinations and circularizations of MDRGI, supporting the possibility of horizontal transfer. Additionally, we have shown in our study that *erm*(B) is not constrained to unique clusters of strains, which can be the case in China, for example. In fact, while *erm*(B) is carried mainly by ST-872, ST-1145, and ST-3753 in China (39), in France, all positive isolates are unique (ST-860, ST-5507, ST-1666, ST-1055, ST-828, and ST-10025).

Although the majority of erythromycin-resistant strains sequenced in our study indeed presented a mutation in the *23S rDNA* sequence, the predominance of *erm*(N) over *erm*(B) is different from what has been reported in other studies previously published, particularly in Asia. Overall, *erm*(N) methyltransferase has rarely been isolated from erythromycin-resistant *Campylobacter* worldwide. In fact, it was reported only in humans in Quebec (Canada) (13) in 2019 and France in 2016 (12). To date, no *erm*(N)-positive isolate has been found among veterinary or food isolates. A possible reason may be that the vast majority of laboratories worldwide prioritize the monitoring of *erm*(B)-expressing and *23S*-mutated isolates, at the expense of newly described mechanisms. Furthermore, the *erm*(N) nucleotide sequence has only recently been added to public resistance databases such as ResFinder and CARD. As previously mentioned, the predominance of *erm*(N) expressed within a chromosomal CRISPR–cas9 operon may also constrain any horizontal transmission of erythromycin resistance to isolates that already display a CRISPR–cas9 operon, as shown in our previous study (12). Associated resistance genes may also include fitness costs for the bacteria, which can explain the predominance of *23S rDNA* mutations in *C. jejuni* (33). These assumptions are, however, inconsistent with the higher and increasing rates of *erm*-positive clinical isolates in France, especially *erm*(N). We are also concerned with the appearance of the first strain described to date to present both a mutation in *23S rDNA* and *erm*(N) methyltransferase (CNRERY-01509). Further investigations are needed to clearly understand *erm*(N) and its diffusion.

As shown here, erythromycin MICs were lower for *erm*(N)-positive isolates than for *erm*(B) or *23S*-mutated isolates. Such a phenotypic approach may, therefore, be considered to monitor the presence of putative *erm*-positive isolates without the use of WGS. The ECDC proposed that high-level resistance to erythromycin (MIC >128 mg/L) could potentially indicate transferable erythromycin resistance due to the presence of the *erm*(B) gene. For *erm*(B)-positive isolates tested by disk diffusion (Table S1), no inhibition zone around the erythromycin disk could be observed (the 6-mm zone equals the disk size). This is not the case for *C. coli* isolates expressing *erm*(N) according to our data, where disk diffusion and MIC values are ranging from 6 to 16 mm and from 16 to ≥256 mg/L, respectively. While erythromycin resistance of our strains remains evident (DD and MIC cutoffs values for *C. jejuni* and *C. coli* based on the CASFM/EUCAST 2022 recommendations are as follows: ≤20 mm and ≥4 mg/L), the risk of overlooking these strains in routine laboratories due to misinterpretation is minimal. The dispersion of erythromycin MIC levels of *erm*(N)-positive isolates remains more visible when assessed by the reference agar dilution technique.

The multiresistant nature of *erm-*positive strains may also be an unusual feature that should attract attention. The resistance profiles according to the resistome identified by WGS in our study favor MDR strains, with an accumulation of genes involved in resistance to aminoglycosides, as already described (39). This finding likely indicates significant selection pressure in animal reservoirs. Source attribution markers indicate that poultry would be the main reservoir for both *C. coli* and *C. jejuni*. Unfortunately, while the surveillance of *erm*(B) and *erm*(N) is routinely performed within poultry and cattle reservoirs at the National Reference Laboratory for Campylobacter (LNR Campylobacter, ANSES, Ploufragan, France), no animal data collected in France have indicated their presence to date.

In general, the appearance and emergence of *erm*-positive strains in France need to be fully investigated. While the first description of *erm*(N) in Quebec was in men who have sex with men (13); this is not the case in France according to our clinical data. In the present study, WGS analyses also revealed that this is not a clonal spread either as we found a variety of STs and CCs. In the future, it would be interesting to study the fitness of *erm*-positive strains versus the *23S rDNA*-mutated strains. If this trend continues, the main mechanism associated with erythromycin resistance in France may be replaced, as it appears to be the case in Asia. Our laboratory continues to investigate all erythromycin-resistant strains *via* next-generation sequencing (NGS) and is encouraging microbiologists in France (human and veterinary) and abroad to use the same strategy for the monitoring of *erm*-positive strains and their associated reservoirs.

## Data Availability

Data is available under ENA project number PRJEB79030. Accession numbers for each genome fasta file are listed in Supp Table 1.

## SUPPLEMENTAL MATERIAL

Table S1 aac.01668-24-s0001.xlsx Complete data table of all French erythromycin-resistant clinical isolates of *C. jejuni* and *C. coli* analyzed in the study.

ASM does not own the copyrights to Supplemental Material that may be linked to, or accessed through, an article. The authors have granted ASM a non-exclusive, world-wide license to publish the Supplemental Material files. Please contact the corresponding author directly for reuse.
